# Impact of the COVID-19 pandemic on referrals and clinical activity in a child and adolescent mental health service in Qatar: a 3-year retrospective comparative analysis

**DOI:** 10.1192/bji.2025.10054

**Published:** 2026-02

**Authors:** Yasser Saeed Khan, Lolwa Kamal, Mohamed Adil Shah Khoodoruth, Sheena Shaheer, Prem Chandra, Mona Mohammad Saleem, Asieh Dehwari, May Jasem Almeraisi, Majid Alabdulla

**Affiliations:** 1 Child and Adolescent Mental Health Service, Hamad Medical Corporation, Doha, Qatar; 2 Medical Research Centre, Hamad Medical Corporation, Doha, Qatar; 3 Mental Health Service, Hamad Medical Corporation, Doha, Qatar; 4 College of Medicine, Qatar University, Doha, Qatar

**Keywords:** Referrals, children and adolescents, mental health, psychiatry, resources

## Abstract

**Background:**

The formative years of childhood and adolescence shape the course of future mental health. The COVID-19 pandemic has been associated with increased mental health problems in young people. This study aimed to examine changes in referrals and clinical activity in a child and adolescent mental health service (CAMHS) in Qatar following the pandemic.

**Aims:**

To explore changes in referral trends and clinical activity in CAMHS, including referral numbers, reasons, sources, demographics, urgency and multidisciplinary team (MDT) allocation, comparing pre-pandemic (2019) with post-pandemic periods (2021, 2022).

**Method:**

A retrospective analysis of referral data from CAMHS was conducted. Data were collected from the administrative paper data archived in the relevant department for the years 2019, 2021 and 2022. Referral data included: source, reason, urgency, patient demographics and outcome. Chi-square analysis was employed to compare referral trends and patient characteristics across the 3 years. Binary logistic regression was used to identify factors associated with urgent referrals.

**Results:**

A significant increase in referrals was observed post-pandemic, with notable changes in referral reasons (increased mood and anxiety disorders), sources (increased referrals from public and private hospitals) and urgency (higher proportion of urgent referrals). MDT allocation shifted towards psychiatrists, with a decrease in joint assessments.

**Conclusions:**

The COVID-19 pandemic had a substantial impact on CAMHS referrals and clinical activity in Qatar. The observed changes highlight the urgent need for additional resources and services. Adapting service delivery models and strengthening collaboration between healthcare sectors are crucial to addressing the evolving mental health needs of children and adolescents effectively.

Childhood and adolescence are critical periods for mental health development.^1^ Approximately one in eight children and young people (CYP) have a diagnosable mental health condition in the UK and an estimated 13.4% of children are affected worldwide.^2^ It is estimated that half of all long-term mental disorders begin by the age of 14, and three-quarters by the age of 24.^1^ Despite this, many young people do not receive the necessary treatment.^3^ The distribution of mental health conditions varies across different demographics, with older children, girls and those from deprived areas more likely to experience poor mental well-being.^4^ These disorders significantly impact the health, education and well-being of CYP and have substantial economic consequences for society.^5,6^ Trends in the UK and across the globe indicate a rising prevalence of mental health problems and persistent inequalities in mental well-being, though detailed and comprehensive data are still lacking.^4^ In recent decades, there has been a marked increase in referrals to out-patient child and adolescent mental health services (CAMHS),^5,7^ yet large unmet needs persist among CYP with mental disorders.^8^

The impact of the COVID-19 pandemic on the mental health of children and adolescents particularly has been studied considerably worldwide.^9–11^ There is evidence emerging that the psychological impact of the pandemic may have contributed to an increase in the prevalence of mental disorders in children and adolescents.^12,13^ Rates of suicidal ideation and suicide attempts in young people have also risen.^14,15^ The impact of the COVID-19 pandemic on the lives of children and adolescents in the state of Qatar has been no different than in the rest of the world. Studies conducted locally have reported increased prevalence rates of mental disorders and pandemic fears in children in the general population, those infected with COVID-19 and adolescents with pre-existing mental disorders.^16,17^

The emerging data related to the effects of the pandemic and its social restrictions on the mental health of children and adolescents is likely to have implications for CAMHS globally.^18^ A study in the Republic of Ireland demonstrated a sharp increase in the number of referrals almost a year after the onset of the COVID-19 pandemic.^19^ This study reported on all referrals from January to November 2020 received by 5 CAMHS in Dublin, which are responsible for a catchment area of 260 560 youth. It found that despite the initial drop in referrals during the early stages of the pandemic, there was a significant increase in the latter part of the year, indicating a rebound effect as restrictions eased. Other studies have also reported a large increase in the treatment demand for children and adolescents with mental health problems.^20,21^ However, data suggesting an actual increase in referrals to CAMHS in Qatar and any change in clinical activity is not currently available. CAMHS faces the challenge of balancing limited resources with growing demands, as investments do not meet the required levels to serve all CYP in need.^22,23^

Addressing unequal access to CAMHS has been a long-standing priority, particularly in the period of transition and recovery from the impacts of COVID-19.^24,25^ Effective planning and allocation of resources within CAMHS require up-to-date knowledge on referral patterns, but international research in this area remains limited.^26^ The population of Qatar was estimated to be around 2.7 million in September 2020 out of which 14.4% (393 000) were aged 0–14 years.^27^ Hamad Medical Corporation (HMC) is the principal public healthcare provider in Qatar. Its CAMHS provides specialist care and support to CYP under 18 years of age with mental and behavioural disorders and their families. This is a community-based service with a multidisciplinary composition with consultant psychiatrists, clinical psychologists, psychiatric nurses, speech and language and occupational therapists, a dietitian and a social worker all working in close liaison. It receives referrals from primary care health centres, clinical departments in secondary and tertiary care public hospitals, private hospitals, schools and social services. Referrals are triaged by a dedicated team of psychiatrists and psychiatric nurses.^28^

## Aims

The primary purpose of this service evaluation study is to explore any changes in referral trends and clinical activity in a specialist outpatient CAMHS in Qatar following the COVID-19 pandemic. The aims of the study are outlined below:

- Examine the total number of referrals received annually and compare pre-pandemic (2019) with post-pandemic periods (2021 and 2022).
- Categorise the reasons and sources of referrals, identifying any significant trends while comparing 2019 with 2021 and 2022.
- Evaluate age, gender and ethnicity of the referred patients, highlighting any notable shifts in the demographic profile over the 3 years.
- Compare the number of urgent and routine referrals allocated for each year along with their rates of acceptance to the service, identifying potential significant shifts.
- Summarise the allocation of referrals to different disciplines within CAMHS, highlighting changes in the allocation patterns and shifts in the types of expertise required.

The year 2020 was an outlier due to significant healthcare disruptions caused by the COVID-19 pandemic. During this period, referral processes were heavily impacted, and many referrals occurred without in-person patient consultations, raising concerns about data reliability. These reasons made 2020 an unsuitable benchmark for comparison with more stable periods.

While the World Health Organization declared the global public health emergency of COVID-19 officially over on 5 May 2023, Qatar began gradually lifting most significant restrictions earlier, starting in 2021. By mid-2022, health services in Qatar had largely resumed normal operations, with only minor preventive measures still in place. Therefore, selecting 2021 and 2022 for our post-pandemic comparison allows for a more accurate assessment of referral trends during a period of more stable healthcare operations in Qatar, following the peak impact of the pandemic’s second wave.

## Method

HMC CAMHS receives referrals from external care providers daily via the Cerner electronic database. The sources of referrals include the primary health care corporation (PHCC), the child development centre (developmental paediatrics) (Peds-CDC), multiple specialties in public general hospitals, private hospitals and schools. Referrals to the service are reviewed by the referrals triage team comprising a consultant psychiatrist, middle-grade psychiatrists and two psychiatric nurses within 24 h of their receipt. If the referral has no adequate clinical information to establish suitability for the service, prompt communication is made with the referring clinician, who is asked to provide the required information.

If the referral is deemed suitable but the information is still insufficient to prioritise and allocate it appropriately, telephone contact is made with the young person and/or their parents to obtain additional information. This process of contacting young people and their families, referred to as screening, mainly serves the purpose of establishing the urgency of referral and identifying the most suitable clinician from the multidisciplinary team (MDT) for allocation. The information gathered during the screening process includes relevant aspects of clinical history and risk issues. Referrals are deemed urgent or routine based on information obtained from the referrer and the patient (or the patient’s family). Referrals deemed urgent are offered appointments for initial assessments in the CAMHS out-patient clinic within 2 weeks, and routine referrals within 6 weeks, of their receipt.

A record of all the received referrals, their source, brief reason for referral, urgency and their outcome with the allocation (if applicable) is captured in weekly minutes by a member of the CAMHS MDT, in addition to being documented on the Cerner clinical information system. This record is shared electronically with all the MDT members weekly to ensure allocation is registered. The paper copy of all referrals with their outcomes is archived securely with the senior executive secretary of the department.

The data, as identified above, were utilised to compare referral trends between the year before the onset of the COVID-19 pandemic (1 January 2019 to 31 December 2019) and the 2 years after the end of the second wave of the pandemic (1 January 2021 to 31 December 2021, and 1 January 2022 to 31 December 2022). The year 2020 was excluded from the comparison as the aim of this study was to compare pre- and post-COVID data, while 2020 represented an outlier period where healthcare disruptions significantly affected referral processes. Additionally, most referrals in 2020 were made without the patient being seen physically, which raises concerns about data reliability. The trend in 2020 is assumed to be one of lower referral numbers due to service disruptions, making it unsuitable for direct comparison with stable pre- and post-pandemic periods.

While the World Health Organization declared the global public health emergency of COVID-19 ended on 5 May 2023, it is important to note that in Qatar, most significant restrictions were gradually lifted starting in 2021. By mid-2022, health services had largely returned to normal operations, though some preventive measures remained in place. The selected comparison periods were chosen to reflect more stable healthcare operations in Qatar post-second wave, ensuring a more accurate assessment of referral trends.

The total number of referrals along with their urgency and sources was compared across the 3 years. The type of referrals – for example, the reason for referral – was also reviewed. The reasons for referrals in our dataset included a wide range of descriptions. For ease of data review and analysis, we classified them into recognised diagnostic categories after making clinical interpretation of the original referral descriptions. This approach allowed for consistency and comparability across cases while maintaining the clinical relevance of the data. The clinical presentations were clubbed together in five major categories, namely (a) mood (b) anxiety (c) neurodevelopmental and behavioural (d) abnormal behaviour and (e) miscellaneous. Symptoms suggestive of obsessive-compulsive disorder or post-traumatic stress disorder were included in ‘anxiety’; those indicating likely psychosis in ‘abnormal behaviour’; and emotional instability, self-harm, depression and hypomania or mania were included in the ‘mood’ group. Attachment-related presentations were included in the ‘neurodevelopmental and behavioural’ category for easier data analysis. These presentations primarily involved young children exhibiting symptoms such as a chronic pattern of emotional withdrawal, rarely seeking comfort when distressed, unexplained irritability, or fearful and sad behaviour, often in the context of severe neglect or inconsistent caregiving. Other less common clinical presentations were included in the ‘miscellaneous’ category.

We also reviewed the demographic characteristics of the patients referred to the service across the 3 years to see if a certain age group or ethnicity was overrepresented in any way. Finally, the outcomes of referrals – for example, acceptance rates for referrals in the 3 years and the MDT allocation of accepted referrals – were also reviewed and compared to identify any potential significant trend changes.

All referrals included in this study were for initial assessments. The dataset does not include information on how many of these referred patients were retained in the service for follow-up and how many were discharged after the initial assessment, although the latter is likely to be a very small number. This study focused on referral trends and allocation rather than long-term patient outcomes. This retrospective review of administrative data did not require ethical approval from the institutional review board; instead, departmental approval was advised and duly obtained.

### Statistical analysis

Descriptive statistics were used to summarise and determine the sample characteristics and distribution of participants’ data. Quantitative data were presented using mean and standard deviation (s.d.). Categorical data were summarised using frequencies and percentages. The chi-square for trend analysis test was employed to examine and statistically evaluate the linear trend in the percentage of referrals. The data were utilised to compare referral trends between the year before the onset of the COVID-19 pandemic (year 2019) and the 2 years after the end of the second wave of the pandemic (years 2021 and 2022). Chi-squared goodness-of-fit analysis was conducted to examine if there was a statistically significant difference between groups, with standard residuals, which indicate the difference between the actual and observed frequencies, helping identify the source. The absolute values >2 show significantly greater deviation than expected, with the plus (+) and minus (−) sign showing the direction of the diversion (+, more; −, less). Associations between two or more qualitative variables across 3 periods were examined and assessed using Pearson’s chi-square or Yates’ corrected chi-square tests as appropriate. Mean age values across 3 periods were compared using one-way analysis of variance (ANOVA) followed by the Bonferroni multiple-comparison test. The key findings were graphically represented using appropriate statistical graphs. Logistic regression method was applied to assess the potential predictors and factors associated with urgent referrals, and the results were presented using odds ratio (OR) values along with respective 95% confidence intervals (CI). All *P*-values presented were two-tailed, and *P*-values <0.05 were considered statistically significant. All statistical analyses were conducted using IBM SPSS Statistics for Windows, version 29.0 (IBM Corp., Armonk, NY, USA), and Epi Info™ for Windows, version 2000 (Centers for Disease Control and Prevention, Atlanta, GA, USA).

## Results

### Annual referral trends

**Fig. 1:**
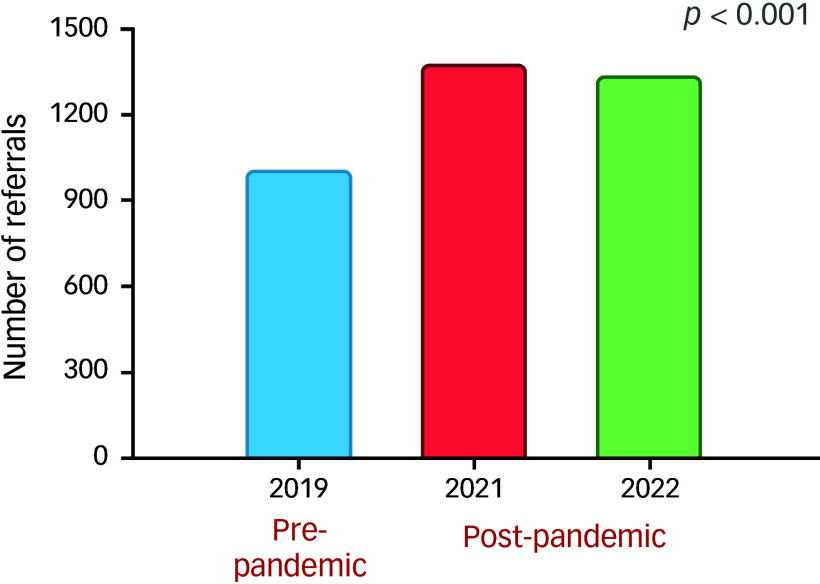
Comparison of total referral numbers before and after the COVID-19 pandemic.

The analysis of annual referral trends revealed a statistically significant increase in the number of referrals to CAMHS following the onset of the COVID-19 pandemic (Fig. 1). Specifically, referrals rose from 1000 in 2019 to 1371 in 2021 before slightly decreasing to 1332 in 2022 (χ^2^ = 67.35, *p* < 0.001) (Table 1).

**Table 1.** Annual referral trends

Referrals	Year 2019	Year 2021	Year 2022	χ^2^ test	***p*** **-value**
Received					
*n*	1000	1371	1332	67.35	<0.001
Standard residual	−6.67	3.89	2.78		
Accepted					
*n*	617 (62.8)	849 (61.9)	798 (59.9)	2.25	0.324
Standard residual	0.6	0.2	−0.7		
Urgent					
*n*	89 (14.4)	228 (26.9)	146 (18.5)	36.98	<0.001
Standard residual	−3.3	4.1	−1.3		

### Reasons for referral

**Fig. 2:**
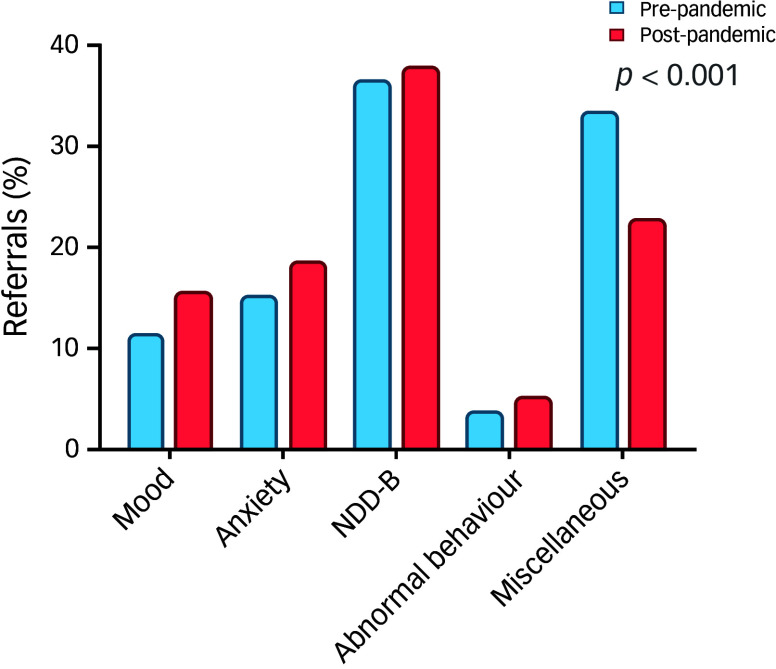
Reasons for referral. NDD-B, neurodevelopmental and behavioural disorders.

The results from the chi-square tests of independence suggest significant changes in the reasons for referral between the years 2019 (pre-pandemic), and 2021 and 2022 combined (post-pandemic) (Fig. 2).

Mood-related referrals increased from 11.4% in 2019 to 15.5% in the post-pandemic period, with a corresponding standardised residual increase from −2.5 to 1.5. Anxiety-related referrals also rose from 15.1 to 18.5%, with standardised residuals moving from −1.9 to 1.1. Referrals for neurodevelopmental disorders showed a slight increase from 36.4 to 38%, with a change in standardised residuals from −0.6 to 0.4. Referrals for abnormal behaviour saw an increase in percentage from 3.7 to 5.2%, though standardised residuals showed a less significant shift from −1.6 to 1.0.

The chi-square test yielded a Pearson’s χ^2^ value of 48.876 (d.f. = 4, *p* < 0.001), indicating a statistically significant difference in the distribution of reasons for referrals across the pre- and post-pandemic periods. Additionally, the linear-by-linear association was significant (*χ*² = 38.922, *p* < 0.001), confirming a significant trend across the years.

### Sources of referral

**Fig. 3:**
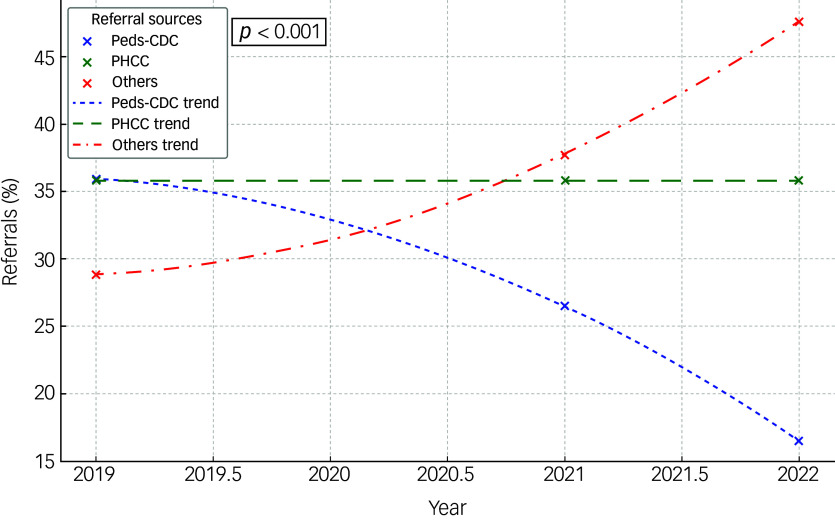
Sources of referral. Peds-CDC, paediatrics, including the child development centre; PHCC, primary health care corporation.

The distribution of referrals from three distinct sources – Peds-CDC, PHCC and other source (public general hospitals, private hospitals, and schools) – were analysed over the years 2019, 2021, and 2022. Peds-CDC accounted for 35.9% of the referrals in 2019, which decreased to 16.5% by 2022. This decline is statistically underscored by a substantial standardised residual in 2022 (−6.2), indicating a significant drop from expected counts. PHCC maintained a consistent level of referral contributions across the 3 years, with percentages closely clustered around the overall average (35.8%). Referrals from other sources saw an increase from 28.8% in 2019 to 47.6% in 2022, with a positive standardised residual of 4.9 in 2022, suggesting a significant increase from expected counts (Fig. 3).

The chi-square test of independence showed a Pearson’s χ^2^ value of 133.708 (d.f. = 4, *p* < 0.001), confirming significant variations in referral sources over the studied years. The linear-by-linear association for the categories showed a significant trend with a statistic of 130.256 (*p* < 0.001), indicating a significant linear trend in the changes in referral sources over the years.

### Demographic characteristics

Table 2 presents the demographic details of the participants across 3 years. There was a statistically significant change in gender distribution over the years. In 2019, females comprised 41% of the sample, which increased to 48% in 2021 (*p* < 0.05) and then slightly decreased to 44% in 2022. The proportion of Qatari nationals decreased from 31.5% in 2019 to 26.6% in 2021 (*p* < 0.05). No significant changes were noted within the Middle East and North Africa (MENA) group, Asian or Others categories across the 3 years.

**Table 2.** Demographic details across 2019, 2021 and 2022

Demographic details, *N* (%)	2019 (*N* = 1000)	2021 (*N* = 1371)	2022 (*N* = 1332)
Mean age (years), ± s.d.	11.4 ± 4.3^a^	11.6 ± 4.0^a^	11.5 ± 3.8^a^
Gender			
Female	410 (41.0)^a^	658 (48.0)^b^	586 (44.0)^a,b^
Male	590 (59.0)^a^	713 (52.0)^b^	746 (56.0)^a,b^
Ethnicity			
Qatari nationals	315 (31.5)^a^	365 (26.6)^b^	372 (27.9)^a,b^
MENA	411 (41.1)^a^	608 (44.3)^a^	567 (42.6)^a^
Asian	178 (17.8)^a^	269 (19.6)^a^	275 (20.6)^a^
Others	96 (9.6)^a^	129 (9.4)^a^	118 (8.9)^a^

Values in the same row and sub-table not sharing the same superscript are significantly different at *p* < 0.05 in the two-sided test of equality for column proportions. Tests assume equal variances. Tests are adjusted for all pairwise comparisons within a row of each innermost sub-table using the Bonferroni correction. MENA, Middle East and North Africa.

### Urgency of referrals

**Fig. 4:**
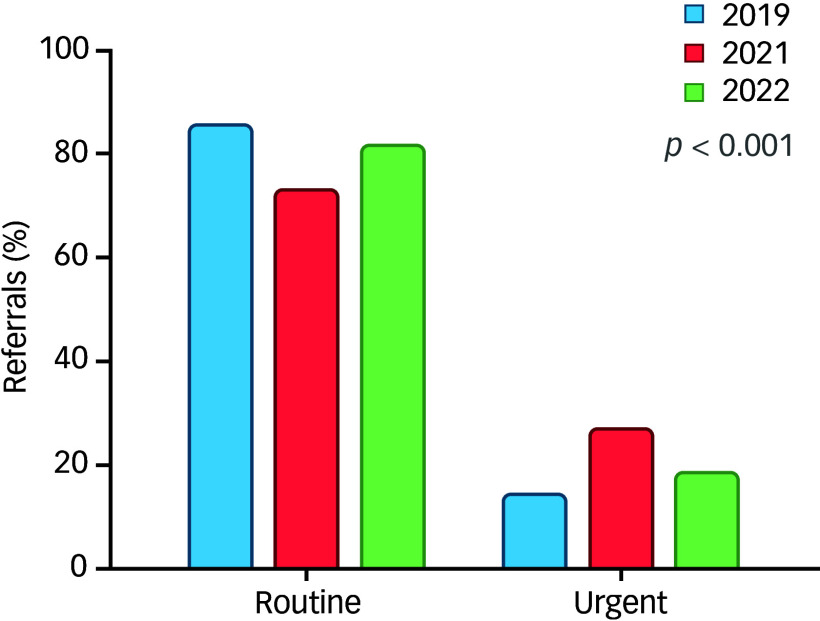
Urgency of referrals.

The data on referrals categorised by urgency (routine versus urgent) were analysed for the years 2019, 2021 and 2022. The proportion of routine referrals fluctuated over the years, accounting for 85.6% in 2019, decreasing to 73.1% in 2021 and rising again to 81.5% in 2022. Standardised residuals indicate that the proportion in 2021 was significantly lower than expected (residual = −2.1). Conversely, urgent referrals constituted 14.4% in 2019, rose to 26.9% in 2021, and then decreased to 18.5% in 2022 (Fig. 4). The standardised residual for 2021 was notably high (residual = 4.1), indicating a significant deviation from expected values.

A chi-square test of independence revealed a Pearson’s χ^2^ value of 36.987 (d.f. = 2, *p* < 0.001), indicating statistically significant differences in referral patterns over the years. The linear-by-linear association for the categories was not significant (χ² = 1.987, *p* = 0.159), suggesting that there is no consistent increasing or decreasing linear trend in the urgency of referrals over the examined period.

A binary logistic regression was conducted to identify factors associated with the urgency of medical referrals. The analysis included variables such as year of referral, age, gender, ethnicity, reason for referral and the source of referral (Table 3).

**Table 3:** Variables associated with the urgency of referrals – logistic binary regression with ‘urgency of referrals’ as the dependent variable

Variable	***B***	s.e.	Wald test	***p*** **-value**	Odds ratio	95% CI for odds ratio (lower)	95% CI for odds ratio (upper)
Year: 2021 versus 2019	0.697	0.154	20.492	<0.001	2.007	1.485	2.714
Year: 2022 versus 2019	0.245	0.165	2.199	0.138	1.277	0.924	1.765
Age (years)	0.036	0.018	4.221	0.040	1.037	1.002	1.073
Gender: female versus male	0.267	0.121	4.902	0.027	1.306	1.031	1.654
Ethnicity: MENA versus Qatari nationals	−0.166	0.14	1.412	0.235	0.847	0.645	1.114
Ethnicity: Asian versus Qatari nationals	−0.24	0.166	2.083	0.149	0.787	0.568	1.09
Ethnicity: others versus Qatari nationals	−0.248	−0.22	1.271	0.260	0.78	0.507	1.201
Reason for referral: anxiety versus mood	−1.54	0.169	83.206	<0.001	0.214	0.154	0.299
Reason for referral: NDD-B versus mood	−1.937	0.174	123.299	<0.001	0.144	0.102	0.203
Reason for referral: abnormal behaviour versus mood	−0.885	0.255	12.061	<0.001	0.413	0.25	0.68
Reason for referral: miscellaneous versus mood	−1.079	0.157	47.447	<0.001	0.34	0.25	0.462
Referral from: PHCC versus Peds-CDC	−0.581	0.161	12.995	<0.001	0.559	0.408	0.767
Referral from: others versus Peds-CDC	0.101	0.149	0.461	0.497	1.107	0.826	1.482
Constant	−0.917	0.302	9.242	0.002	0.40		

MENA, Middle East and North Africa; NDD-B, neurodevelopmental and behavioural disorders; PHCC, primary health care corporation; Peds-CDC, paediatrics, including the child development centre.

Referrals in 2021 were significantly more likely to be urgent compared to those in 2019 (odds ratio 2.007, 95% CI [1.485, 2.714], *p* < 0.001). Each additional year of age was associated with a higher likelihood of a referral being urgent (odds ratio 1.037, 95% CI [1.002, 1.073], *p* = 0.040). Female gender was associated with higher odds of an urgent referral compared to males (odds ratio 1.306, 95% CI [1.031, 1.654], *p* = 0.027). Referrals for anxiety were significantly less likely to be urgent compared to those for mood disorders (odds ratio 0.214, 95% CI [0.154, 0.299], *p* < 0.001). Similarly, referrals for neurodevelopmental disorders and behavioural disorders (odds ratio 0.144, 95% CI [0.102, 0.203], *p* < 0.001) and abnormal behaviour (odds ratio 0.413, 95% CI [0.250, 0.680], *p* < 0.001) were less likely to be urgent compared to mood. Miscellaneous reasons also showed significantly lower odds of urgency (odds ratio 0.340, 95% CI [0.250, 0.462], *p* < 0.001). Therefore, mood disorders stand out as a category where referrals were more likely to be urgent. Referrals from PHCC were significantly less likely to be urgent compared to those from Peds-CDC (odds ratio 0.559, 95% CI [0.408, 0.767], *p* < 0.001).

### MDT allocation

The distribution of MDT resources was analysed across 3 years. The roles were categorised as follows: (a) psychiatric doctors, (b) psychiatric nurses, (c) psychologists, (d) allied health professionals and (e) joint assessments.

**Fig. 5:**
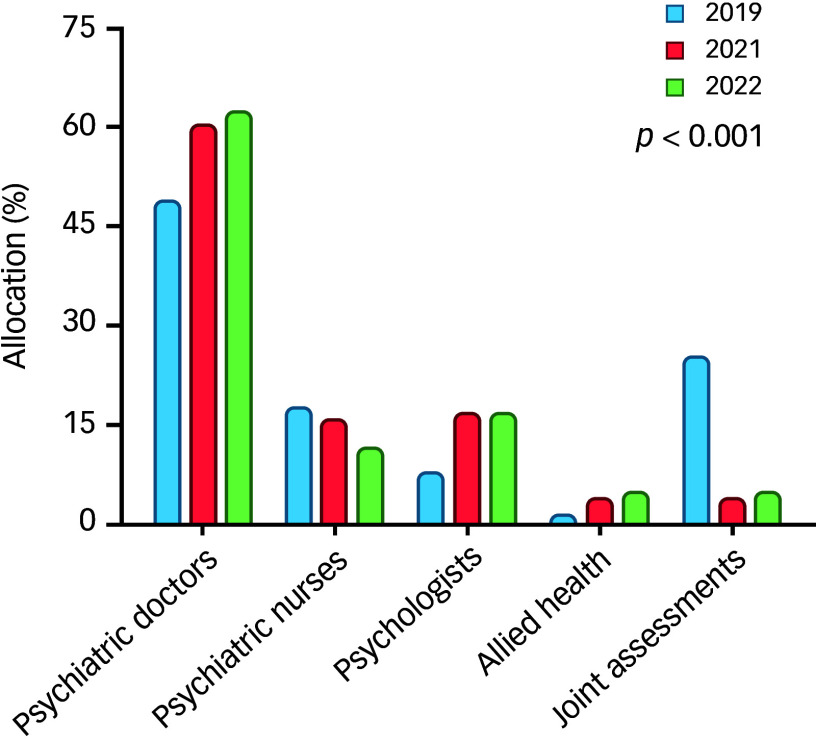
Multidisciplinary team (MDT) allocation.

The allocation to psychiatrists increased from 48.8% in 2019 to 62.6% in 2022, with a standardised residual increasing over time. Psychiatric nurses experienced a decrease from 17.3% in 2019 to 11.4% in 2022, with a negative residual in the latest year, indicating a reduction in their proportional involvement. The allocation to psychologists increased by more than twofold from 7.6% in 2019 to approximately 16.7% in 2022 with allocation to allied health professionals also seeing an increase from 1.1% in 2019 to 4.7% in 2022. Joint assessments by different disciplines in the team, which involve collaborative efforts among various specialists, decreased dramatically from 25.1% in 2019 to 4.7% in 2022, with a notably high positive residual in 2019, followed by significant negative residuals, suggesting a major shift (Fig. 5).

A chi-square test of independence yielded a Pearson’s χ^2^ value of 261.633 (d.f. = 8, *p* < 0.001, indicating significant differences in the MDT allocation patterns across the 3 years. Additionally, the linear-by-linear Association was significant (*χ*² = 62.962, *p* < 0.001), indicating a significant trend across the years.

## Discussion

The findings from our study reveal significant changes in referral patterns, reasons for referral, sources of referral, demographic characteristics, urgency of referrals and MDT allocation to CAMHS before and after the onset of the COVID-19 pandemic. The notable increase in annual referrals from 1000 in 2019 to 1371 in 2021, followed by a slight decrease to 1332 in 2022, stresses the heightened demand for CAMHS during and immediately after the pandemic. This trend aligns with global reports indicating increased mental health issues among children and adolescents due to the pandemic’s disruption of daily life, education and social interactions.^29,30^ The increase in referrals might not solely reflect new cases of mental health concerns; changes in referral practices within schools or paediatric care facilities, or simply increased awareness of mental health issues among parents and educators, could also contribute to this trend. A study aimed to investigate the impact of the COVID-19 pandemic on out-of-hours presentations to CAMHS in North Central and East London.^31^ The analysis showed a significant drop in CAMHS presentations during the pandemic, particularly from March to July 2020, with fewer telephone consultations and on-site assessments conducted. This decline suggests that children and families were hesitant to seek mental health support due to fears of contracting the virus. Consequently, this reluctance might have contributed to a surge in referrals and presentations post-pandemic as delayed mental health issues emerged. These findings underscore the importance of ensuring accessible and safe mental health support during crises to prevent future surges in demand.

The shift in reasons for referral, with significant increases in mood-related and anxiety-related referrals, underscores the mental health impact of the pandemic. The rise in mood-related referrals from 11.4% in 2019 to 15.5% in the post-pandemic period and anxiety-related referrals from 15.1 to 18.5% is consistent with studies documenting elevated levels of depression and anxiety among young people during the pandemic.^32^ The pandemic might have exacerbated pre-existing vulnerabilities,^33^ with school closures and social isolation particularly impacting young people who rely on these structures for emotional support and a sense of belonging.^34,35^ Additionally, increased family stress due to job insecurity or financial strain could have created a more challenging home environment, contributing to mood and anxiety disorders.^36^ The stable yet slightly increased referrals for neurodevelopmental disorders and abnormal behaviour reflect ongoing needs in these areas, further compounded by pandemic-related stressors.

The changing patterns in referral sources, with a significant decrease in referrals from Peds-CDC, and an increase in referrals from other sources such as public and private hospitals and schools, indicate a broader engagement of different sectors in addressing mental health needs. The substantial drop in Peds-CDC referrals from 35.9% in 2019 to 16.5% in 2022 and the rise in referrals from other sources from 28.8 to 47.6% suggest that schools and general hospitals are increasingly recognising and responding to mental health issues, a positive shift towards more comprehensive care. The reasons behind the decrease in referrals from Peds-CDC are unclear and require further investigation.

The changes in demographic characteristics, particularly the increase in female referrals and the decrease in referrals from Qatari nationals, reflect broader societal and cultural shifts. The increase in female referrals from 41% in 2019 to 48% in 2021 may indicate greater recognition and reporting of mental health issues among females, who have been found to experience higher rates of anxiety and depression in general^37,38^ and during the pandemic.^39,40^ The decrease in Qatari national referrals could be due to various factors, including access to private mental health services, cultural stigma or potentially increased social support compared to expatriate populations, rendering them more resilient. Future research could explore specific reasons for the reduction in referrals for Qatari nationals.

The fluctuations in the urgency of referrals paint a concerning picture of the pandemic’s impact on mental health crises among young people. The peak in urgent referrals in 2021 (26.9%) compared to 2019 (14.4%) and 2022 (18.5%) suggests that the pandemic’s initial disruptions and uncertainties may have triggered a surge in acute mental health needs. This aligns with reports of increased rates of self-harm and suicidal ideation among young people during the pandemic.^41,42^ The binary logistic regression analysis provided valuable insights into factors associated with urgent referrals. While the year of referral (2021) was a significant predictor, other factors like age, gender and reason for referral also played a role, indicating that the pandemic’s impact wasn’t homogenous and certain groups of young people may have been more vulnerable to experiencing mental health crises.

The shortage of specialist mental health services for children and adolescents was recognised before the pandemic.^43,44^ The increased prevalence rates of mental health problems in this special population since the onset of the pandemic and the consequent escalation in referrals have clearly put further pressure on existing services. The HMC CAMHS service in Qatar has adapted at an unprecedented pace to manage the increased demand since the onset of the pandemic, mainly due to the robust triage process ensuring appropriate prioritisation and allocation of referrals. However, steps need to be taken both locally and internationally to alleviate the escalating pressure on CAMHS services worldwide.

The observed changes in MDT allocation patterns may be explained by multiple factors. The shift towards greater psychiatrist involvement and reduced joint assessments likely reflects a change in service delivery strategies post-pandemic. The increase in referrals with complex mental health concerns may have necessitated a higher proportion of psychiatric evaluations. Additionally, resource reallocation within CAMHS in response to increased demand may have influenced the distribution of referrals among MDT members. The observed reduction in the allocation to psychiatric nurses’ initial assessment clinics may be attributed to several factors. Firstly, psychiatric nurses have increasingly taken on additional responsibilities beyond their initial assessment clinics. These include playing a more active role in the triage of new referrals alongside senior psychiatrists and participating in specialised clinics, such as those for consultation and autism spectrum disorder assessments. This expanded scope of duties highlights the evolving and multifaceted role of psychiatric nurses within the healthcare system.

Our findings are consistent with international studies reporting an increase in CAMHS referrals post-pandemic. A study in the Republic of Ireland showed a similar surge in referrals one year after the onset of COVID-19, with heightened demand persisting beyond initial lockdowns.^19^ A survey of mental health professionals in Switzerland also found that treatment demand for children and adolescents increased significantly after an initial drop during the early lockdown period, with many professionals reporting service overload, longer waiting times and insufficient resources to meet the heightened demand.^20^

Further supporting this trend, a recent study examining general practitioner referrals to a Dublin-based CAMHS found that post-pandemic, there was a rise in referrals for anxiety, self-harm and eating disorders, while referrals for psychosis and autism spectrum disorder declined.^45^ However, studies directly comparing pre- and post-pandemic trends in referrals to specialist CAMHS remain scarce. Our study contributes to this gap by providing data on how referral patterns, urgency and service demands evolved in a specialist CAMHS setting, offering valuable insights for future service planning. Together, these international studies suggest that the increased mental health burden on children and adolescents observed in our study is part of a broader global trend, reflecting changes in referral patterns and the types of mental health concerns presenting to CAMHS worldwide.

This study’s strength lies in its ability to inform policy and resource allocation decisions for CAMHS. The need for additional resources and potential changes in operational strategies can be better assessed based on the study’s findings. Collaborative efforts between healthcare professionals, policymakers and researchers are essential to devise strategies that not only address the increased demand but also ensure the sustainability and effectiveness of CAMHS services in the post-pandemic era.

In conclusion, this study highlights the noteworthy surge in referrals to Qatar’s HMC outpatient CAMHS during and after the COVID-19 pandemic. The implications extend beyond Qatar’s borders, echoing global concerns about the pandemic’s impact on the mental health of young individuals. The data generated by this study has significant implications for refining the strategies of CAMHS in Qatar and beyond, ensuring that the needs of this vulnerable population are adequately met. The measures required in the short and medium term include the provision of additional much-needed resources to the already strained specialist secondary care CAMHS teams for enhancement of recruitment and setting up appropriate community and in-patient facilities. There is a need for more emphasis on effective triage of referrals for better utilisation of resources and effective care delivery. The innovative practice of conducting telepsychiatry consultations (phone or video) for certain patients is likely to assist in reducing the no-show rate and avoiding long waiting lists. The role of crisis resolution and home treatment teams can be particularly crucial in managing acute and severe presentations requiring intensive monitoring in the community, thereby reducing the load on regular outpatient services.

In the long term, the focus should be placed on prevention strategies. All professionals working with children and adolescents need to establish effective communication channels between their organisations to ensure help can be sought from the appropriate service in a timely manner. These mainly include CAMHS, paediatrics, primary healthcare practitioners, schools and social services. Awareness programmes focusing on the reduction of stigma, education on early signs of mental disorders and how to seek help promptly need to be held regularly.

### Limitations and future research

As with other studies, ours has potential limitations. First, it lacks detailed qualitative data, which might have provided in-depth insights into the reasons behind changes in referral patterns and the experiences of children and families. Additionally, the retrospective design of the study tends to introduce potential biases. Finally, the geographical restriction to Qatar may limit the generalisability of the findings to other regions. Further research adopting qualitative methods and a broader geographical scope would be valuable for a more comprehensive understanding of the pandemic’s impact on CAMHS services.

While the inclusion of formal ICD-11 or DSM-5 diagnoses could deepen the analysis, our study was specifically designed to explore changes in referral content and symptoms based on the information provided in referrals. This approach allowed us to identify emerging patterns in symptoms and signs, helping us to better understand the immediate impact of the pandemic on mental health services. However, we recognise that incorporating formal diagnoses could provide additional perspectives. Doing so would require a different study design and more resources, which were beyond the scope of this analysis. Future research could build on our findings by integrating formal diagnostic data to further explore the long-term effects of the pandemic on mental health services.

## Data Availability

Data can be made available upon request from the corresponding author.
